# Quality of Life in Patients with Pancreatic Cancer before and during the COVID-19 Pandemic

**DOI:** 10.3390/ijerph19063731

**Published:** 2022-03-21

**Authors:** Andrea Alexander, Stephen Fung, Martin Eichler, Nadja Lehwald-Tywuschik, Vasuki Uthayakumar, Sami-Alexander Safi, Christian Vay, Hany Ashmawy, Sinan Kalmuk, Alexander Rehders, Sascha Vaghiri, Wolfram Trudo Knoefel

**Affiliations:** 1 Department of General, Visceral and Pediatric Surgery, University Hospital Düsseldorf and Medical Faculty, Heinrich-Heine-University Düsseldorf, 40225 Duesseldorf, Germany; andrea.alexander@med.uni-duesseldorf.de (A.A.); stephen.fung@med.uni-duesseldorf.de (S.F.); nadja.lehwald-tywuschik@med.uni-duesseldorf.de (N.L.-T.); vasuki.uthayakumar@med.uni-duesseldorf.de (V.U.); sami-alexander.safi@med.uni-duesseldorf.de (S.-A.S.); christian.vay@med.uni-duesseldorf.de (C.V.); hany.ashmawy@med.uni-duesseldorf.de (H.A.); sinan.kalmuk@med.uni-duesseldorf.de (S.K.); rehders@med.uni-duesseldorf.de (A.R.); 2 Clinic and Polyclinic for Internal Medicine I, University Hospital Carl Gustav Carus Dresden, 01307 Dresden, Germany; martin.eichler@uniklinikum-dresden.de

**Keywords:** quality of life, pandemic, COVID-19, pancreatic tumor

## Abstract

Background: Coronavirus disease 19 (COVID-19) substantially affects cancer patients due to adverse outcomes and disruptions in cancer care. Recent studies have indicated the additional stress and anxiety burden arising from the pandemic and impairing quality of life in this vulnerable group of patients. However, patients with cancer represent a heterogenous group. Therefore, we conducted a study on patients with pancreatic cancer, requiring demanding surgical interventions and chemotherapy regimens due to its aggressive tumor biology, to explore the pandemic’s impact on quality of life within this homogenous cohort. Methods: In a descriptive observational study, the quality of life of patients who had undergone pancreatic surgery for tumor resection at our institution between 2014 and the beginning of the pandemic in March 2020 was assessed. For HRQoL measurement, we used the European Organisation for Research and Treatment of Cancer Quality of Life Core Questionnaire (EORTC QLQ-C30), comparing their situation before the pandemic and since its beginning. An additional self-developed questionnaire was applied to assess the life circumstances during the pandemic. Results: Our cohort included 26 patients. Scores from the survey in HRQoL revealed no significant changes over time between before and during the pandemic. A medium deterioration in HRQoL was apparent in social functioning, as well as a small deterioration in role functioning and emotional functioning. Worries concerning a potential impact of COVID-19 on personal health were expressed. Psychological limitations in QoL were mainly attributed to the pandemic, whereas physical limitations in QoL were rather associated with the underlying disease of pancreatic cancer. Conclusion: The COVID-19 pandemic is causing considerable social and emotional distress among pancreatic cancer patients. These patients will benefit from psychological support during the pandemic and beyond. Long-time survivors of pancreatic cancer, such as those included in our cohort, appear to have improved resilience facing the psychosocial challenges of the pandemic. For pancreatic cancer, surgical care is considered the cornerstone of treatment. Prolonged delays in healthcare cause serious damage to mental and physical health. To date, the longer-term clinical consequences are not known and can only be estimated. The potential tragic outcome for the vulnerable group of pancreatic cancer patients highlights the urgency of timely healthcare decisions to be addressed in the future.

## 1. Introduction

The rapidly expanding coronavirus disease 2019 (COVID-19) caused by the severe acute respiratory syndrome coronavirus 2 (SARS-CoV-2), was first identified in China in December 2019 and declared as a pandemic by the World Health Organization (WHO) on 11 March 2020 [1]. Regarded as a serious global health threat, a variety of measures have been implemented worldwide to limit the spreading of the virus. Since avoiding human-to-human exposure and limiting social contacts are essential in this matter, these measures have impacted most aspects of daily life resulting in negative effects on psychological wellbeing [2].

The ongoing pandemic and repetitive lockdowns have led to a sense of fear and anxiety around the globe [3,4]. It is no surprise that enforced isolation on the one hand and increasing fear of severe illness due to COVID-19 on the other may impair quality of life [5]. Beyond basic measures of physical and mental health the multidimensional concept of health-related quality of life encompasses medical and social perceptions [6].

Therefore, as well as monitoring the incidence rates, it is crucial to assess the psychological and social impact of the disease and the implemented restrictions [7].

Regarding these aspects, patients with cancer have been affected to a great extent by the COVID-19 pandemic [8,9]. As well as enduring psychosocial distress, cancer patients are confronted with two additional issues.

Firstly, there are findings of adverse outcomes in patients with cancer who develop COVID-19. Due to the immunosuppressive effect of malignancies and the surgical procedures, chemotherapeutics and radiation applied to treat them, as well as more exposure to the virus in healthcare facilities, cancer patients appear to be more susceptible to SARS-CoV-2 infections. Data from China, Europe and the United States showed a higher mortality rate in patients with cancer than in patients without cancer [10,11,12,13]. 

Secondly, massive disruptions in cancer care due to overwhelmed healthcare facilities have led to delays in cancer therapy—which render this group of people particularly vulnerable. As well as progression of the disease if untreated, Gagliardi et al. showed that waiting for postponed procedures may cause depression or anxiety in cancer patients, adversely affecting mental health and spoiling trust in the healthcare system [14].

Several studies have demonstrated an impairment of quality of life caused by the pandemic in cancer patients collectively [15,16,17,18] as well as for particular entities such as patients with sarcomas [19] or thyroid cancer [20]. The effect on patients with pancreatic cancer has not been investigated yet. 

Due to its aggressive tumor biology, pancreatic cancer is often diagnosed at an advanced stage. Medical or surgical treatment remains demanding for the patients, and any treatment carries a substantial percentage of adverse effects [21]. 

Forasmuch as the continuation of oncological care is essential for the complex therapy of pancreatic cancer, it is of our special interest to investigate the quality of life in these patients in the context of the pandemic situation [22,23].

Therefore, the present study aims to assess the effect of the COVID-19 pandemic on the quality of life of patients with pancreatic cancer.

## 2. Methods

We conducted a single-center cross-sectional descriptive observational study. All 35 living patients who had undergone pancreatic surgery for tumor resection at our institution between 2014 and the beginning of pandemic restrictions in March 2020 were contacted by phone in July 2021 and asked to participate. Patients who were unavailable or mentally or linguistically unable to complete the questionnaires were excluded.

Surgical procedures included partial pancreatoduodenectomy, distal and total pancreatectomy, as well as enucleation and duodenal preserving partial pancreatectomy. Partial pancreatoduodenectomy was usually performed as a pylorus-preserving procedure with a three-loop reconstruction. Lymphadenectomy routinely included clearance of the peripancreatic, hepatoduodenal, celiac and interaortocaval lymph nodes. All procedures were carried out by a transverse laparotomy. 

All collected data were adhered to the guidelines established by the Declaration of Helsinki. Patients being followed up by our staff were contacted by telephone, informed about the study and asked to consent. They were then asked to complete the questionnaires regarding the quality of their lives before and since the beginning of the pandemic. Clinical information was obtained from medical records.

For HRQoL measurement, we used the European Organisation for Research and Treatment of Cancer Quality of Life Core Questionnaire (EORTC QLQ-C30) [24]. This instrument measures in units from 0 to 100, global quality of life and 5 functioning and 9 symptom domains, where high values indicate better HRQoL (functioning domains) and higher symptom burden (symptom domains), respectively. The patients gave two answers to each question—regarding their situation before the pandemic and since its beginning. 

To evaluate the strength of clinical meaningful differences we used the evidence-based guidelines for interpreting EORTC-C30 chance scores developed by Cocks et al. [25].

We used a self-developed questionnaire to assess the life circumstances of patients during the pandemic. This questionnaire consists of 17 questions: (1) potential delays in tumor-aftercare, (2) cancellations of medical appointments by the patients themselves, (3) current chemotherapeutic treatment, (4) impairment of quality of life during the pandemic, (5) concerns regarding COVID-19 infection, (6) concerns regarding severe COVID-19 disease course, (7) vaccination status, (8) improvement in HRQoL after vaccination, (9) financial problems, (10) increased burden during the pandemic, (11) better mastering of the pandemic due to increased resilience, (12) felt restrictions due to the lockdown measures, (13) behaviour during lockdown, (14) behaviour during lockdown in relation to the underlying condition, (15) limitations in quality of life, (16) cause of these limitations, (17) particular experiences, (18) additional comments.

Since the answers to these questions were quite varying and highly individual, we used mixed methods to describe the resulting aspects. Seventeen questions were analysed with absolute and relative frequencies; 2 questions were evaluated qualitatively. 

Clinical data were collected from patients’ medical records, compiled into an Excel-file database and analyzed retrospectively. We obtained the following data from medical records: demographic parameters (age, gender), tumor characteristics (histology), treatment characteristics (time since diagnosis, surgical procedures, chemotherapy, disease and treatment status).

## 3. Results

### 3.1. Participation and Sample Description

Thirty-one patients were approached and 26 participated in the study. All patients who participated completed both questionnaires. Our patient collective consisted of 12 female and 14 male patients, the median age was 65 (range 20–82 years). Patient and tumor characteristics are summarized in Table 1. 

Thirteen patients underwent partial pancreatoduodenectomy, ten patients had distal pancreatectomy, one patient required total pancreatectomy, one patient received an enucleation and one patient had a duodenal preserving partial pancreatectomy.

Twelve of the patients had an adenocarcinoma, 12 had neuroendocrine tumors, whereas 2 patients had a pseudopapillary carcinoma.

All patients were assessed by a multidisciplinary tumor board consisting of oncologic surgeons, oncologists, radiotherapists, pathologists and radiologists. According to the recommendation of the tumor board, 12 patients received adjuvant chemotherapy, whereas 10 of these had finished chemotherapy when surveyed.

### 3.2. Health-Related Quality of Life before and during the Pandemic

**Figure 1:**
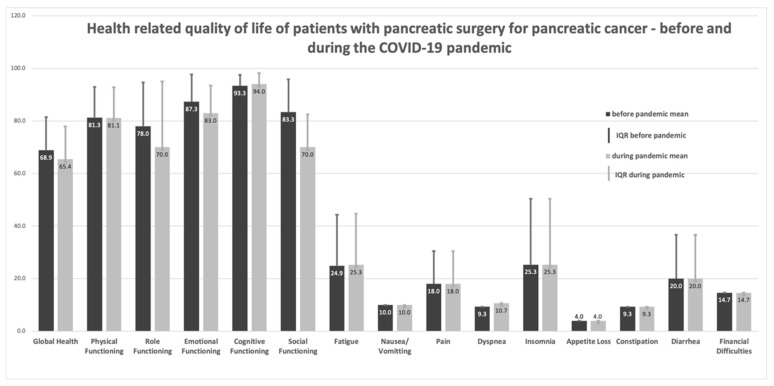
Health-related quality of life of patients with pancreatic surgery for pancreatic cancer—before and during the COVID-19 pandemic. Measurement: EORTC QLQ C-30. Small deteriorations: emotional functioning, role functioning. Medium deterioration: social functioning. All other differences: trivial (according to Cocks 2012).

Mean global HRQoL was 68.9 out of a maximum of 100 points before, and 65.3 after the begin of the pandemic. Among the functioning scales, the highest decline was apparent in social functioning (83.3 to 70) (medium deterioration according to Cocks). Role functioning (78 to 70) and emotional functioning (87.3 to 83) also showed a deterioration during the pandemic (small deterioration). Differences in physical functioning, the symptom domains and all other domains were trivial or non-existent (Figure 1). Most of the patients reported clinically important limitations such as occasional weakness or gastrointestinal problems.

However, there was no significant effect of the pandemic situation on these symptoms. Figure 1 shows the respective results.

### 3.3. Self-Developed Questionnaire

Data from the survey of our self-developed questionnaire to assess the life circumstances of patients with pancreatic cancer during the pandemic are shown in Table 2. In addition, the patients had the opportunity to include free text comments.

The majority of patients (24/26) had no delay in regular tumor patient follow-up due to clinical capacity restrictions of the hospital. However, some patients (5/26) cancelled their follow-up appointments themselves because of fear of infection. In fact, more than half of the patients worried considerably about becoming infected with SARS-CoV-2, whereas 23% additionally worried about having a severe course of the disease if infected. Almost all (96%) patients are now fully vaccinated against COVID-19 (July 2021). Interestingly, only about half of the patients (48%) reported having an improved quality of life after vaccination. Clearly, most patients did not feel additionally burdened by the pandemic (85%) when compared to their condition before. More than two-thirds (69%) did not even feel restricted due to the lockdown measures. When asked whether they felt they might master the corona crisis better than others due to the resilience they had developed, 15 patients agreed.

Twenty-three of the patients adhered strictly to lockdown regulations regarding staying at home and reducing social contacts; 11 of them did so due to their underlying condition making them more vulnerable. Twenty-four of all patients stated to have physical or psychological limitations in their quality of life. Of these, 14 patients had physical limitations—in most cases (13) due to their disease. In contrast, out of the 18 patients having psychological limitations in quality of life, 14 felt this was because of the pandemic. 

Qualitatively evaluated comments revealed interesting remarks. Summarizing, many of the patients seemed to be affected by the current situation caused by the pandemic. One patient reported her grandchildren ran away from her because of fear of infecting her. Another did not dare to go on vacation due to the pandemic. Another patient needs urgent surgery because of severe adhesions but refuses to have surgery now because of fear of infection and visitor restrictions in the hospitals. One other patient still wears gloves everywhere due to fear of infection. Several patients reported having increasing financial problems, partially because they cannot work as much as before due to the disease—however, the addition of the pandemic situation seems to have made this more difficult.

## 4. Discussion

Previous studies have revealed a wide range of psychosocial effects of the COVID-19 pandemic on cancer patients [15,16,26,27,28]. Cancer patients are already subjected to considerable physical and emotional stress related to their disease and are widely known to be at increased hazard for a severe affection by COVID-19 [29,30]. Global health status, changes in healthcare trajectories, stress and anxiety related to the coronavirus, a decrease in physical and virtual contact with family and friends, general quality of life, emotional functioning and pain scores were all effected to some extent in one of the previous studies on cancer patients. Many of the effects were, however, not significant. Additional significant changes in these parameters in comparison to levels before the pandemic were not necessarily found at all timepoints [28]. 

Cognitive and social functioning were significantly reduced in one of these studies including more than 200 patients. In contrast to our study, this report included patients under chemotherapy with different oncological entities [15]. We could not confirm these significant changes. Cognitive functioning was unaffected and there was a trend towards an impairment in social functioning. Some of this difference may be attributed to the different sample size. More likely, the current administration of chemotherapy exerted an effect on these psychosocial parameters. 

Cancer patients’ increased susceptibility to complicated infections has been emphasized by Liang et. al, reporting an up to 3.5-fold increase in the risk of needing mechanical ventilation or ICU admission in the case of SARS-CoV2 infection compared to patients without cancer. This study included patients with different cancers. Lung cancer was the most frequent diagnosis. [10]. 

For patients with pancreatic cancer, no data have been reported on this issue thus far. To the best of our knowledge, this is the first study addressing the impact of the COVID-19 pandemic on the quality of life of patients with pancreatic cancer. All patients had undergone resection in curative intent. Two of the 26 patients enrolled were still receiving adjuvant chemotherapy. All patients required structured follow-up. None of the patients were in tier 1 or tier 3 (high or low priority level) according to the ESMO recommendations [31]. Since a delay in follow-up for more than 8 weeks may result in a poorer outcome, especially early after surgery, due to a delay in adequate therapy, we classified all patients into tier 2. Since patients are educated to strictly observe the given follow-up rules, the inability to comply with these rules will not only pose a potential risk for health but also increase the disease-related stress level. Furthermore, patients will have to weigh the need for medical treatment against the risk of infection before every consultation. This additional burden may influence HRQoL.

Interestingly, in our cohort, scores from the present survey in HRQoL revealed no significant changes over time comparing health-related quality of life before and during the pandemic. Similar findings have been previously described [16,20], where no major effects on QoL by the added stress of the COVID-19 pandemic were revealed. This might be a result of an increased resilience in particular experienced by long-time cancer survivors, as found in our cohort [32,33]. However, overall, many of the patients with pancreatic cancer in our cohort seem to be affected by the current situation caused by the pandemic in several ways. The concerns expressed in the free comments were individual and rather complex to categorize. No trend to a leading complaint or a specific burden was detected.

A medium deterioration in HRQoL [25] was apparent in social functioning, as well as a small deterioration in role functioning and emotional functioning. Worries concerning a potential impact of COVID-19 on personal health were expressed. Psychological limitations in QoL were mainly attributed to the pandemic, whereas physical limitations in QoL were rather associated with the underlying disease of pancreatic cancer.

Although worse outcomes have been documented for COVID-19 in men, higher levels of concern of obtaining an infection were reported by women in our cohort (66% of women vs. 42% of men). This confirms previously reported data that women seem to have experienced more anxiety, fears, stress and depression during the pandemic [30,34].

### 4.1. Perspectives

The data collected in this survey indicate an emotional burden of our patients caused by the COVID-19 pandemic. Individual statements highlight the value of experiences with major challenges.

In challenging times like these, listening to the concerns of our patients and learning from their experience to ensure a medical care to the best of our ability is essential. Flexibility and precautions taken during the pandemic using digital resources to monitor pancreatic cancer patients to minimize attendance and therefore reducing the risk of infection, may be implemented [18]. Certainly, pancreatic cancer patients will benefit from psychological support during the pandemic and beyond.

### 4.2. Future Aspects

Our cohort of patients had surgery before the beginning of the pandemic, and most of the patients who required chemotherapy had finished the therapy by then. Thus, they were merely affected by delays in treatment in the setting of the COVID-19 pandemic. 

However, to avoid healthcare system exhaustion, many healthcare facilities throughout the world have delayed and are still delaying elective surgical procedures including those for cancer [35,36]. Prolonged waiting for procedures may have a serious impact on mental health [14]. For pancreatic cancer, surgical care is considered the cornerstone of treatment [22]. There are data indicating that the discontinuation of oncological care during the pandemic may result in additional loss of lives since some patients may not receive adequate surgical treatment in time [22,36,37,38]. The potential tragic outcome for this vulnerable group of patients highlights the urgency of timely healthcare decisions to be addressed in the future.

### 4.3. Study Limitations

The limitations of our study include the handling of the EORTC QOL-C30 questionnaire, which is not designed to be used for retrospective surveys. A comparison of a retrospective and prospective application of another quality of life questionnaire yielded a high agreement [39]. This questionnaire has been used in a setting very comparable to ours [40]. This may suggest a potential use of the EORTC QLQ-C30 in our setting. Measuring change in quality of life by retrospective evaluation remains a matter of current discussion. [41]. Our intent was to uniquely collect data interrogating the same cohort of patients concerning their QoL both before and during the COVID-19 pandemic, rendering a valid comparison. Other studies have compared EORTC QOL-C30 with reference values from cancer patients obtained under non-pandemic conditions by EORTC [15,19], or with non-pandemic normative population values if not available for the investigated tumor type [19]. Since different cancer types are very heterogenous in terms of tumor biology, clinical behavior and responsiveness to treatment, such data could be biased. Emphasizing the investigation of a homogenous group of pancreatic cancer patients, we accepted possible shortcomings due to false memory. Moreover, we added a pandemic-specific self-developed questionnaire. Long-time survivors of pancreatic cancer represent a rare homogenous cohort. Therefore, we decided to conduct this study despite the small number of included patients and used mixed methods of description.

## 5. Conclusions

The COVID-19 pandemic is causing considerable social and emotional distress among pancreatic cancer patients regardless of their current healthcare needs. To date, the longer term clinical consequences are not known and can only be estimated. 

However, the COVID-19-related disruption in cancer care causes anxiety and psychological burdens due to the pandemic itself, as well as to constant delays of procedures and resulting uncertainty. Moreover, any delay in cancer treatment may result in tumor progress from being curable to becoming non-curable. Future studies are needed to focus on pancreatic cancer patients treated during the COVID-19 pandemic to ensure high-quality medical care.

## Figures and Tables

**Table 1:** Description of study population.

Variable	Value	N = 26 (%)
Sex	female	12 (46.2)
	male	14 (53.6)
Age at study inclusion	mean	65
	18–40	5 (19.2)
	41–55	3 (11.5)
	56–65	8 (30.8)
	66–75	5 (19.2)
	>75	5 (19.2)
Time since diagnosis	1 and <2 years	3 (11.5)
	2–5 years	13 (50)
	more than 5 years	9 (34.6)
Tumor Type	Adenocarcinoma	12 (46.2)
	Neuroendocrine Tumor	12 (46.2)
	Pseudopapillary Carcinoma	2 (7.7)
Staging		
Adenocarcinoma	IA	0 (0)
	IB	3 (11.5)
	IIA	3 (11.5)
	IIB	6 (23.1)
	III	0 (0)
NET	I	7 (26.9)
	IIA	0 (0)
	IIB	2 (7.7)
	IIIA	0 (0)
	IIIB	3 (11.5)
Staging (PC)	T3N0M0	2 (7.7)
Type of Surgery	Whipple	13 (50)
	Distal pancreatic resection	10 (38.5)
	Enucleation	1 (3.9)
	Pancreatectomy	1 (3.9)
	Duodenal preserving partial pancreatectomy	1 (3.9)
Chemotherapy received	Yes	12 (46.2)
	No	14 (53.9)

**Table 2:** Self-developed Questionnaire. Management of the COVID-19 pandemic in a sample of pancreatic cancer patients.

Questions N = 26	YES N (%)	NO N (%)
Follow-up was postponed due to pandemic?	2 (7.7)	24 (92.3)
Did you cancel any medical appointments due to fear of infection?	5 (19.2)	21 (80.8)
Are you in current chemotherapeutic treatment?	2 (26.9)	24 (73.1)
Does your quality of life seem to be impaired due to the pandemic?	10 (38.5)	16 (61.5)
Did you worry a lot about getting infected with SARS-Cov-2?	14 (53.8) Female 8 Male 6	12 (46.2) Female 4 Male 8
Did you worry a lot about having a severe course of a SARS-Cov-2 infection?	6 (23.1)	20 (76.9)
Are you vaccinated?	25 (96.0)	1 (4.0)
Did your vaccination improve your quality of life?	12 (48.0)	13 (52.0)
Do you have increasing financial problems?	4 (15.4)	22 (84.6)
Do you feel burdened due to the pandemic—more than before?	4 (15.4)	22 (84.6)
Do you feel that you may master the corona crisis better than others due to more resilience developed during your disease?	15 (57.7)	11 (42.3)
Do you feel restricted due to lockdown measures?	8 (30.8)	18 (69.2)
How did you behave during the lockdown regarding leaving home and social contacts	as required	rather casual
	23 (88.5)	3 (11.5)
Did you adhere more strictly to the measures due to your underlying condition?	YES N (%)	NO N (%)
	11 (42.3)	15 (57.7)
Do you have physical or psychological quality of life limitations?	24 (92.3)	2 (7.7)
Do you have physical quality of life limitations?	14 (53.8)	12 (46.2)
If so—do do you attribute them more to your disease and surgery or to the pandemic?	Disease	Pandemic
	13 (92.9)	1 (7.1)
Do you have psychological quality of life limitations?	YES N (%)	NO N (%)
	18 (69.2)	8 (30.8)
If so—do do you attribute them more to your disease and surgery or to the pandemic?	Disease	Pandemic
	4 (22.2)	14 (77.8)

## Data Availability

The data presented in this study are available on request from the corresponding author. The data are not publicly available due to the very individual nature of the freetext answers.

## Abbreviations

COVID-19	Coronavirus disease 2019
SARS-CoV-2	Severe acute respiratory syndrome coronavirus 2
WHO	World Health Organization
QoL	Quality of Life
EORTC QLQ-C30	European Organisation for Research and Treatment of Cancer Quality of Life Core Questionnaire
HRQoL	Health-Related Quality of Life

## References

[1] Ghebreyesus T.A., World Health Organization (2020). WHO Director-General’s Opening Remarks at the Media Briefing on COVID-19-11 March 2020.

[2] Holmes E.A., O’Connor R.C., Perry V.H., Tracey I., Wessely S., Arseneault L., Ballard C., Christensen H., Silver R.C., Everall I. (2020). Multidisciplinary research priorities for the COVID-19 pandemic: A call for action for mental health science. Lancet Psychiatry.

[3] Lehmann J., Holzner B., Giesinger J.M., Bottomley A., Ansari S., von Butler L., Kemmler G. (2021). Functional health and symptoms in Spain before and during the COVID-19 pandemic. BMC Public Health.

[4] Peteet J.R. (2020). COVID-19 Anxiety. J. Religion Health.

[5] Horn L., Garassino M. (2020). COVID-19 in patients with cancer: Managing a pandemic within a pandemic. Nat. Rev. Clin. Oncol..

[6] Hennessy C.H., Moriarty D.G., Zack M.M., Scherr P., Brackbill R. (1994). Measuring health-related quality of life for public health surveillance. Public Health Rep..

[7] Guven D.C., Sahin T.K., Aktepe O.H., Yildirim H.C., Aksoy S., Kilickap S. (2020). Perspectives, Knowledge, and Fears of Cancer Patients About COVID-19. Front. Oncol..

[8] Frasquilho D., Matias R., Grácio J., Sousa B., Luís-Ferreira F., Leal J., Cardoso F., Oliveira-Maia A. (2021). Protocol for the Implementation and Assessment of “MoodUP”: A Stepped Care Model Assisted by a Digital Platform to Accelerate Access to Mental Health Care for Cancer Patients Amid the COVID-19 Pandemic. Int. J. Environ. Res. Public Health.

[9] Moraliyage H., De Silva D., Ranasinghe W., Adikari A., Alahakoon D., Prasad R., Lawrentschuk N., Bolton D. (2020). Cancer in Lockdown: Impact of the COVID-19 Pandemic on Patients with Cancer. Oncologist.

[10] Liang W., Guan W., Chen R., Wang W., Li J., Xu K., Li C., Ai Q., Lu W., Liang H. (2020). Cancer patients in SARS-CoV-2 infection: A nationwide analysis in China. Lancet Oncol..

[11] Mehta V., Goel S., Kabarriti R., Cole D., Goldfinger M., Acuna-Villaorduna A., Pradhan K., Thota R., Reissman S., Sparano J.A. (2020). Case Fatality Rate of Cancer Patients with COVID-19 in a New York Hospital System. Cancer Discov..

[12] Onder G., Rezza G., Brusaferro S. (2020). Case-Fatality Rate and Characteristics of Patients Dying in Relation to COVID-19 in Italy. JAMA.

[13] Rogado J., Obispo B., Pangua C., Serrano-Montero G., Marino A.M., Pérez-Pérez M., López-Alfonso A., Gullón P., Lara M.Á. (2020). Covid-19 transmission, outcome and associated risk factors in cancer patients at the first month of the pandemic in a Spanish hospital in Madrid. Clin. Transl. Oncol..

[14] Gagliardi A.R., Yip C.Y.Y., Irish J., Wright F.C., Rubin B., Ross H., Green R., Abbey S., McAndrews M.P., Stewart D.E. (2021). The psychological burden of waiting for procedures and patient-centred strategies that could support the mental health of wait-listed patients and caregivers during the COVID-19 pandemic: A scoping review. Health Expect..

[15] Ciążyńska M., Pabianek M., Szczepaniak K., Ułańska M., Skibińska M., Owczarek W., Narbutt J., Lesiak A. (2020). Quality of life of cancer patients during coronavirus disease (COVID-19) pandemic. Psycho-Oncology.

[16] Baffert K.-A., Darbas T., Lebrun-Ly V., Pestre-Munier J., Peyramaure C., Descours C., Mondoly M., Latrouite S., Bignon E., Nicouleau S. (2021). Quality of Life of Patients With Cancer During the COVID-19 Pandemic. Vivo.

[17] Wang Y., Duan Z., Ma Z., Mao Y., Li X., Wilson A., Qin H., Ou J., Peng K., Zhou F. (2020). Epidemiology of mental health problems among patients with cancer during COVID-19 pandemic. Transl. Psychiatry.

[18] Jeppesen S.S., Bentsen K.K., Jørgensen T.L., Holm H.S., Holst-Christensen L., Tarpgaard L.S., Dahlrot R.H., Eckhoff L. (2021). Quality of life in patients with cancer during the COVID-19 pandemic—A Danish cross-sectional study (COPICADS). Acta Oncol..

[19] Younger E., Smrke A., Lidington E., Farag S., Ingley K., Chopra N., Maleddu A., Augustin Y., Merry E., Wilson R. (2020). Health-Related Quality of Life and Experiences of Sarcoma Patients during the COVID-19 Pandemic. Cancers.

[20] Falcone R., Grani G., Ramundo V., Melcarne R., Giacomelli L., Filetti S., Durante C. (2020). Cancer Care during COVID-19 Era: The Quality of Life of Patients With Thyroid Malignancies. Front. Oncol..

[21] Catanese S., Pentheroudakis G., Douillard J.-Y., Lordick F. (2020). ESMO Management and treatment adapted recommendations in the COVID-19 era: Pancreatic Cancer. ESMO Open.

[22] Oba A., Stoop T.F., Löhr M., Hackert T., Zyromski N., Nealon W.H., Unno M., Schulick R.D., Al-Musawi M.H., Wu W. (2020). Global Survey on Pancreatic Surgery During the COVID-19 Pandemic. Ann. Surg..

[23] Ueda M., Martins R., Hendrie P.C., Mc Donnell T., Crews J.R., Wong T.L., McCreery B., Jagels B., Crane A., Byrd D.R. (2020). Managing Cancer Care During the COVID-19 Pandemic: Agility and Collaboration Toward a Common Goal. J. Natl. Compr. Cancer Netw..

[24] Aaronson N.K., Ahmedzai S., Bergman B., Bullinger M., Cull A., Duez N.J., Filiberti A., Flechtner H., Fleishman S.B., De Haes J.C.J.M. (1993). The European Organization for Research and Treatment of Cancer QLQ-C30: A Quality-of-Life Instrument for Use in International Clinical Trials in Oncology. J. Natl. Cancer Inst..

[25] Cocks K., King M.T., Velikova G., de Castro G., St-James M.M., Fayers P.M., Brown J.M. (2012). Evidence-based guidelines for interpreting change scores for the European Organisation for the Research and Treatment of Cancer Quality of Life Questionnaire Core 30. Eur. J. Cancer.

[26] Bakouny Z., Hawley J.E., Choueiri T.K., Peters S., Rini B.I., Warner J.L., Painter C.A. (2020). COVID-19 and Cancer: Current Challenges and Perspectives. Cancer Cell.

[27] Al-Quteimat O.M., Amer A.M. (2020). The Impact of the COVID-19 Pandemic on Cancer Patients. Am. J. Clin. Oncol..

[28] Bartels M.M.T.J., Gal R., van der Velden J.M., Verhoeff J.J.C., Verlaan J.J., Verkooijen H.M. (2021). Impact of the COVID-19 pandemic on quality of life and emotional wellbeing in patients with bone metastases treated with radiotherapy: A prospective cohort study. Clin. Exp. Metastasis.

[29] Wang C., Pan R., Wan X., Tan Y., Xu L., Ho C.S., Ho R.C. (2020). Immediate Psychological Responses and Associated Factors during the Initial Stage of the 2019 Coronavirus Disease (COVID-19) Epidemic among the General Population in China. Int. J. Environ. Res. Public Health.

[30] Kang L., Ma S., Chen M., Yang J., Wang Y., Li R., Yao L., Bai H., Cai Z., Yang B.X. (2020). Impact on mental health and perceptions of psychological care among medical and nursing staff in Wuhan during the 2019 novel coronavirus disease outbreak: A cross-sectional study. Brain Behav. Immun..

[31] Passaro A., Addeo A., Von Garnier C., Blackhall F., Planchard D., Felip E., Dziadziuszko R., de Marinis F., Reck M., Bouchaab H. (2020). ESMO Management and treatment adapted recommendations in the COVID-19 era: Lung cancer. ESMO Open.

[32] Seiler A., Jenewein J. (2019). Resilience in Cancer Patients. Front. Psychiatry.

[33] Macía P., Barranco M., Gorbeña S., Iraurgi I. (2020). Expression of resilience, coping and quality of life in people with cancer. PLoS ONE.

[34] Liu N., Zhang F., Wei C., Jia Y., Shang Z., Sun L., Wu L., Sun Z., Zhou Y., Wang Y. (2020). Prevalence and predictors of PTSS during COVID-19 outbreak in China hardest-hit areas: Gender differences matter. Psychiatry Res..

[35] Larson D.W., El Aziz M.A.A., Mandrekar J.N. (2020). How Many Lives Will Delay of Colon Cancer Surgery Cost During the COVID-19 Pandemic? An Analysis Based on the US National Cancer Database. Mayo Clin. Proc..

[36] Fligor S.C., Wang S., Allar B.G., Tsikis S.T., Ore A.S., Whitlock A.E., Calvillo-Ortiz R., Arndt K.R., Gangadharan S.P., Callery M.P. (2020). Gastrointestinal Malignancies and the COVID-19 Pandemic: Evidence-Based Triage to Surgery. J. Gastrointest. Surg..

[37] Sud A., Torr B., Jones M., Broggio J., Scott S., Loveday C., Garrett A., Gronthoud F., Nicol D.L., Jhanji S. (2020). Effect of delays in the 2-week-wait cancer referral pathway during the COVID-19 pandemic on cancer survival in the UK: A modelling study. Lancet Oncol..

[38] Madan A., Siglin J., Khan A. (2020). Comprehensive review of implications of COVID-19 on clinical outcomes of cancer patients and management of solid tumors during the pandemic. Cancer Med..

[39] Lawson A., Tan A.C., Naylor J., Harris I.A. (2020). Is retrospective assessment of health-related quality of life valid?. BMC Musculoskelet. Disord..

[40] Walle-Hansen M.M., Ranhoff A.H., Mellingsæter M., Wang-Hansen M.S., Myrstad M. (2021). Health-related quality of life, functional decline, and long-term mortality in older patients following hospitalisation due to COVID-19. BMC Geriatr..

[41] Blome C., Augustin M. (2015). Measuring Change in Quality of Life: Bias in Prospective and Retrospective Evaluation. Value Health.

